# DNA-PK contributes to the phosphorylation of AIRE: Importance in transcriptional activity

**DOI:** 10.1016/j.bbamcr.2007.09.003

**Published:** 2008-01

**Authors:** Ingrid Liiv, Ana Rebane, Tõnis Org, Mario Saare, Julia Maslovskaja, Kai Kisand, Erkki Juronen, Leena Valmu, Matthew James Bottomley, Nisse Kalkkinen, Pärt Peterson

**Affiliations:** a Molecular Pathology, University of Tartu, Tartu 50411, Estonia; b Human Biology and Genetics, University of Tartu, Tartu 50411, Estonia; c Institute of Biotechnology, University of Helsinki, Helsinki, Finland; d Istituto di Ricerche di Biologia Molecolare P. Angeletti, Via Pontina Km. 30.600, 00040 Pomezia (Rome), Italy; e Institute of Medical Technology, University of Tampere, Tampere 33014, Finland

**Keywords:** APECED, HSR domain, PHD finger, Phosphorylation

## Abstract

The autoimmune regulator (AIRE) protein is a key mediator of the central tolerance for tissue specific antigens and is involved in transcriptional control of many antigens in thymic medullary epithelial cells (mTEC). Mutations in the *AIRE* gene cause a rare disease named autoimmune polyendocrinopathy-candidiasis-ectodermal dystrophy (APECED). Here we report using GST pull-down assay, mass-spectrometry and co-immunoprecipitation that a heterotrimeric complex of DNA-Dependent Protein Kinase (DNA-PK), consisting of Ku70, Ku80 and DNA-PK catalytic subunit (DNA-PKcs), is a novel interaction partner for AIRE. *In vitro* phosphorylation assays show that the residues Thr68 and Ser156 are DNA-PK phosphorylation sites in AIRE. In addition, we demonstrate that DNA-PKcs is expressed in AIRE positive mTEC cell population and that introduction of mutations into the AIRE phosphorylation sites decrease the capacity of AIRE to activate transcription from reporter promoters. In conclusion, our results suggest that phosphorylation of the AIRE protein at Thr68 and Ser156 by DNA-PK influences AIRE transactivation ability and might have impact on other aspects of the functional regulation of the AIRE protein.

## 1 Introduction

*AIRE* (autoimmune regulator) is a defective gene in APECED (autoimmune-polyendocrinopathy-candidiasis-ectodermal dystrophy, OMIM #240300), the genetic autoimmune disease, which manifests as autoimmunity to multiple endocrine glands. In human tissues, AIRE is expressed in the thymus, spleen, and lymph nodes [1–3].

The AIRE protein (57.5 kDa) has several features which indicate that it might function in transcriptional regulation. AIRE shares similar domain architecture with the Sp100 family proteins (Sp140, Sp110 and Sp100C), which all have HSR (for homogenously staining region), SAND (for Sp100, AIRE-1, NucP41/75 and DEAF-1) and PHD (for plant homeodomain) domains. HSR is central for AIRE di- or oligomerization [4,5]. The SAND domain is a DNA binding domain found in several mammalian nuclear proteins. NMR spectroscopy studies of the Sp100B SAND domain have shown that a conserved amino acid motif KDWK is essential for DNA recognition [6]. Although AIRE lacks the KDWK motif, its SAND domain has also been shown to bind DNA *in vitro* [4]. The PHD fingers are found in nuclear proteins that are often involved in chromatin-mediated transcriptional regulation and appear to mediate protein–protein interactions. The zinc-binding cysteine-rich regions of the PHD finger form two flexible loops and two protein ligands have been proposed to bind one PHD finger simultaneously [7]. It has been suggested that one of the ligands can be specific for each PHD. For example, the PHD finger of ING2 can bind phosphoinositides whereas PHD of the KAP-1 repressor binds to Mi2α, a component of the NuRD chromatin remodelling complex [8,9]. Another ligand, which might be common for all PHD fingers, has been long suggested to be chromatin, and recently, PHD domains of ING2 and BPTF proteins were reported to bind tri-methylated lysine 4 of histone H3 [10,11]. However, despite the determination of its 3-D structure, no conclusive ligand of the AIRE PHD finger has yet been identified [12].

We and others have shown that AIRE can activate reporter genes from the interferon beta promoter or in a system where AIRE is tethered via heterologous GAL4 DNA binding domain to the reporter [5,13]. The transcriptional activation region of AIRE has been initially mapped to the two C-terminal PHD fingers as the mutations in these domains severely decreased the transactivation capacity [13,14]. More recent reports have shown that several other APECED-causing mutations in the HSR or SAND domain also decrease the AIRE transactivation ability [15,16].

So far, the only known protein partner for AIRE is a general transcriptional co-regulator and histone acetyltransferase, CREBP binding protein (CBP). CBP has been reported to bind directly to AIRE *in vitro*, it colocalizes with AIRE into the nuclear bodies in monocytic cell cultures and it enhances the transcription of reporter genes in collaboration with AIRE [5,16]. The mechanisms how AIRE in collaboration with CBP activates its target genes are largely unknown.

DNA-dependent protein kinase (DNA-PK) is a Ser/Thr kinase that belongs to the phosphatidylinositol 3-kinase (PI3K) family. It is formed as holoenzyme in the presence of DNA by two Ku regulatory subunits, Ku70 (70 kDa) and Ku80 (86 kDa), and a large catalytic subunit DNA-PKcs (465 kDa). With very few exceptions, DNA-PKcs is active only when it is in hetero-trimeric complex with Ku proteins and in interaction with DNA or RNA [17,18]. The main function of DNA-PK is to recognize double stranded DNA breaks and to catalyze a repair process known as non-homologous end joining (NHEJ). In a similar way, DNA-PK is crucial for V(D)J recombination in developing T and B cells. Concordantly, DNA-PKcs or Ku deficient mice are severely immunodeficient, with elevated radiosensitivity and susceptible for tumor development [17,19].

In addition to the role of DNA-PK in chromatin repair, DNA-PK has been shown to phosphorylate many proteins involved in cell cycling and transcriptional regulation, such as p53, Sp1, Myc, Fos, Jun, TBP, TFIIB, RNA Polymerase II [17] and several nuclear receptors, for example, the glucocorticoid [20], progesterone [21] and androgen receptors [22]. DNA-PK can positively and negatively modulate transcription. As a repressor, DNA-PK binds directly to a specific DNA sequence element within the mouse mammary tumor virus (MMTV) promoter [20,23]. Similarly, the whole DNA-PK complex has been shown to bind to the E-box/TATA like elements and to suppress the human xanthine oxidoreductase (*hXOR*) gene expression [24]. At the same time, the Ku proteins have been described as transcriptional recycling co-activators of androgen receptor [22]. In addition to these examples, DNA-PK can modulate gene expression via RNA-dependent phosphorylation of hnRNP proteins [18].

In order to further define molecular mechanisms of AIRE functioning, we searched for novel protein partners of AIRE using the GST pull-down approach combined with mass spectrometry. We found that AIRE interacts with DNA-PK complex proteins DNA-PKcs, Ku70 and Ku80. Further *in vitro* phosphorylation assays demonstrated that DNA-PK phosphorylates the AIRE protein *in vitro* at the residues Thr68 and Ser156. In addition, we show that the mutation of either of the phosphorylation sites significantly decreases AIRE transcriptional activity.

## 2 Materials and methods

### 2.1 Plasmids

Plasmid pGST-PHD1 was generated by insertion of PCR-amplified sequence encoding human AIRE-1 amino acids 293–354 into the *Nco*I and *Kpn*I restriction sites of the pETM-30 vector (EMBL-Heidelberg protein expression and purification laboratory). To generate pGST-AIRE-SPP (SAND/PHD1/PHD2), the sequence encoding AIRE amino acids 178–482 was cloned into *Eco*RI and *Xho*I sites of pGEX1λT-SH3 vector (a gift from K. Saksela, University of Tampere, Finland). pGST AIRE-T68A, pGST-AIRE-S156A and pGST-AIRE-V80L mutation constructs were generated by altering amino acid Thr68 or Ser156 to alanines and V80 to leucine in pGST-AIRE [5] constructs by PCR-based site-directed mutagenesis. The control plasmid pGEX-2T-p53 was a gift from T. Punga (Uppsala University, Sweden). pSI-AIRE-T68A and pSI-AIRE-S156A were prepared by further cloning the mutated AIRE fragments from pGST-AIRE-T68A and pGST-AIRE-S156A into *Eco*RI and *Sal*I sites of pSI vector (Promega). To generate luciferase reporter plasmids pBL-INV and pBL-LOR, the promoter areas of involucrin (3737 nt) and loricrin (2217 nt) genes were cut out from the plasmids pTZhINV-nlbgal and pTZhLOR1 (gifts from A. Männik, FitBiotech, Estonia) and cloned into the *Hin*dIII site of pBL-KS (a gift from K. Saksela, University of Tampere, Finland). The cloned plasmids were verified by sequencing.

### 2.2 Expression and purification of GST fusion proteins

The GST-tagged proteins were expressed either from Section 2.1. or previously described [5] AIRE GST-fusion constructs, pGST-AIRE (1–545), pGST-R257X (1–256), pGST-AIRE293 (1–293), pGST-AIRE348 (1–348), pAIRE-SAND (175–298) in *Escherichia coli* BL21-DE3 strain by induction with 0.4 mM IPTG for 4 h at room temperature. Full-length GST-AIRE and PHD-containing fusion proteins were expressed in the presence of 0.1 mM ZnCl_2_. The proteins were purified using Glutathione Sepharose 4B (AmershamBiosciences) according to the manufacturer's instructions except that 1% N-laurylsarcosine and 3% Triton X-100 were added to increase solubility of the proteins and 50 μM ZnCl_2_ was included when GST-AIRE and PHD-containing fusion proteins were purified. The purified proteins were verified on SDS-PAGE and Coomassie Blue staining.

### 2.3 GST pull-down and mass spectrometry

50–100 μg of nuclear extract, prepared from human monocyte cell line THP-1 according to [25], was incubated with 20–25 μg of GST-fusion proteins bound to 25 μl of packed sepharose beads in buffer B1 (10 mM HEPES pH 8.0, 150 mM NaCl, 0.7 mM MgCl_2_, 12.5% glycerol, 0.1 mM EDTA, 25 μM ZnCl_2_, 0.5 mM DTT and proteinase inhibitor mix) over-night at 4 °C. The beads were washed intensively with buffer B1, the bound proteins were eluted and separated on SDS-PAGE. The specific protein bands were cut out and analyzed with mass spectrometry.

Mass mapping of the peptides generated was performed with an Ultraflex™MALDI-TOF/TOF mass spectrometer (Bruker-Daltonics, Bremen, Germany) equipped with a nitrogen laser in a positive ion reflector mode using á-cyano-4-hydroxycinnamic acid as the matrix. The MALDI spectra were externally calibrated with the standard peptide mixture from Bruker-Daltonics (Bremen, Germany). In case of a peptide fragmentation analysis, a peptide from above mass mapping analysis was selected as a precursor ion and subjected for further MS/MS fragmentation in the MALDI-TOF/TOF lift-mode. Database searches were carried out by either Mascot peptide map fingerprint or Mascot MS/MS ions search (http://www.matrixscience.com/).

### 2.4 Transfections and immunoprecipitations

The transfections were carried out with ExGen500 *in vitro* reagent (Fermentas) according to the manufacturer's protocol. For immunoprecipitations, approximately 2 × 10^6^ HEK293 cells were transfected with 10 μg of pcAIRE [3] or either pcDNA3.1B-myc/his (Invitrogen) or pdEYFP-N1 (Clontech) vector. After 46 h, a whole-cell extract was prepared by lysis with 0.25 ml of lysis buffer (20 mM Tris pH 7.5, 1% NP-40, 0.3 M NaCl, 0.1 mM ZnCl_2_, 1.5 mM MgCl_2_, 0.5 mM DTT, 0.2 mM EDTA, 25% glycerol, protease inhibitors) for 30 min on ice. The lysates were treated five times with the 25G syringe to shear genomic DNA, centrifuged at 13 500 rpm, 15 min at 4 °C. The supernatants were diluted 5 times with the lysis buffer lacking NP-40 and immunoprecipitations were performed with mouse monoclonal anti-DNA-PK 4F10C5 (BD Pharmingen), anti-AIRE 6.1 [3], anti-HuR 3A2 (a gift from J. Steitz, Yale University, USA), anti-HA sc-7392 (SantaCruzBiotech) anti-GAPDH 6C5 (Ambion), anti-c-myc 9E10 (Sigma) and rabbit polyclonal anti-GST sc-459 (SantaCruzBiotech) antibodies. 1.5 μg of each antibody was added to 700–900 μg of whole-cell extracts and incubated for 4 h at 4 °C, then 15 μl of packed protein G-Sepharose beads (Amersham Pharmacia Biotech) were included and incubation was continued for 1 h at 4 °C. The immunoprecipitates were washed 4 times with the IP buffer (20 mM Tris pH 7.5, 0.2% NP-40, 0.3 M NaCl, 0.1 mM ZnCl_2_, 1.5 mM MgCl_2_, 0.5 mM DTT, 0.2 mM EDTA, 25% glycerol, protease inhibitors) and eluted from the beads with 2× SDS loading buffer, separated on SDS-PAGE and subjected to Western blot with anti-AIRE 6.1 or DNA-PK antibody (1:1000) and ECL detection. Transfer DNA-PK was carried out with 0.01 M CAPS buffer, pH 11.0, supplemented with 20% methanol in semi-dry blotting system for 90 min at 1 mA/cm^2^.

Where indicated, RNaseA/RNaseT mix (Fermentas) with final concentrations 4 μg/ml and 10 u/ml, respectively, was added. Ethidium bromide (EtBr) and micrococcal nuclease (MNase) treatments were performed as demonstrated earlier [26]. Either 0.4 mg/ml EtBr was added to the extract or 1 u of MNase (Fermentas) in 50 μl of digestion buffer (10 mM HEPES pH 7.0, 4 mM CaCl_2_, 50 mM NaCl, 0.1 mM ZnCl_2_) was used. Digestion efficiency of MNase was tested in the separate control experiments, using the same conditions. On agarose gels, only the mononucleosomal fraction of DNA was detected when whole nuclei were treated and no DNA was left when free DNA was digested.

### 2.5 Computer prediction of AIRE phosphorylation

Scansite 2.0 program at http://scansite.mit.edu/cgi-bin/motifscan_seq [27] and NetPhos 2.0 program at http://www.cbs.dtu.dk/services/NetPhos [28] were used to predict AIRE phosphorylation sites.

### 2.6 Phosphorylation assays

The kinase assays were performed with SignaTECT DNA-PK Kinase Assay System kit (Promega) essentially according to the manufacturer's protocol. The phosphorylation reactions were carried out in the presence of γATP-^32^P for 7 min at 30 °C and either 25 μg of biotinylated-p53 peptide (EPPLSQEAFADLWKK, Promega) or 4–5 μg of GST fusion proteins were used as the substrates. Efficiencies of ^32^P labeling of the substrates were measured by radioactivity counter (1414 Guardian, Wallac). Nuclear extracts and immunoprecipitations were made as described in Sections 2.3 and 2.4. Either 600 μg (Fig. 3A) or 15–20 μg (all other figures) of nuclear extracts were used per reaction. Alternatively, 10 U of purified DNA-PK complex (Promega) in the presence of 250 ng of calf thymus DNA was used. 50 μM LY294002 hydrochloride (Sigma) was added when indicated. In assays using GST fusion proteins as the substrates, the GST proteins bound to 10 μl of packed glutathione beads were pre-washed with the kinase reaction buffer (SignaTECT kit buffer supplemented with 0.1 mM ZnCl_2_ and 0.2 mM Na_3_VO_4_). After the kinase reaction, the beads were washed three times with ice cold washing buffer (25 mM HEPES pH 7.5, 150 mM NaCl, 0.1% BSA, 0.2% NP-40, 0.1 mM ZnCl_2_ and 0.2 mM Na_3_VO_4_) before measuring the radioactivity.

### 2.7 Mice, thymic stromal cell isolation, cell sorting and RT-PCR

C57BL/6J background wild type mice were maintained at the mouse facility of the Institute of Molecular and Cell Biology, Tartu University. Thymuses from 4- to 6-week-old mice were used. Thymic stromal cell isolation, cell sorting and RT-PCR were carried out as described previously [29].

### 2.8 Luciferase reporter assays

Luciferase assays were performed using Luciferase Assay System kit (Promega) according to the manufacturer's protocol. 4 × 10^4^ HEK293 cells in 24-well plates were transfected with 0.1 μg of INV-pBL or LOR-pBL and 0.3 μg pSI-AIRE, pSI-AIRE-T68A or pSI-AIRE-S156A for 46 h. In Western blot, anti-AIRE 6.1 antibody was use and 0.3 μg of pSI-AIRE, pSI-AIRE-T68A or pSI-AIRE-S156A were transfected for 46 h.

## 3 Results

### 3.1 DNA-PK complex proteins co-purify with AIRE PHD domain

**Fig. 1:**
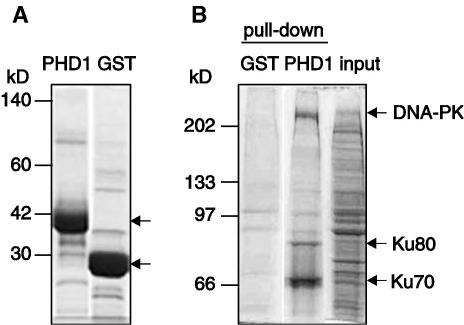
AIRE PHD1 interacts with Ku70, Ku 80 and DNA-PKcs. (A) Coomassie staining of purified GST-AIRE-PHD1 and GST, indicated by arrows. (B) Affinity purification of AIRE-PHD1 binding proteins from THP-1 nuclear extract. Three protein bands that specifically bound PHD1 and that were analyzed by MALDI-TOF are indicated by arrows. Input represents 5% of the amount of nuclear extract used in pull-down.

In order to find new AIRE interacting partners, we expressed the first PHD domain (PHD1) of AIRE as a GST-fusion protein (Fig. 1A) and performed the GST pull-down using the nuclear extract prepared from monocytic THP-1 cells. The interacting proteins were separated on SDS-PAGE and visualized by Coomassie staining. Three protein bands with molecular weight of about 70, 80 and over 250 kDa were consistently seen after the SDS-PAGE analyses (Fig. 1B). Next, the protein bands were excised from the gel and analyzed by mass spectrometry. The MALDI-TOF mass map analysis demonstrated that the two bands with molecular masses of about 70 and 80 kDa belong to Ku70 and Ku80, respectively, whereas the protein with the mass over 250 kDa was presumed to be the catalytic subunit of DNA-PK (DNA-PKcs) (Table 1). Mass map identifications with low sequence coverage were further verified by MALDI-TOF/TOF fragmentation of three tryptic peptides (Table 1). Thus, these results identified a trimeric complex of DNA-PK as a candidate AIRE interacting partner.

### 3.2 Full-length AIRE interacts with DNA-PK

**Fig. 2:**
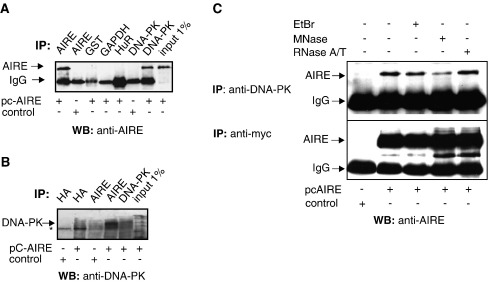
DNA-PK co-immunoprecipitates AIRE from cell lysates. (A, B) Whole-cell extracts were prepared either from AIRE or control transfected HEK293 cells, and used in co-immunoprecipitations as indicated. Antibodies that were applied in co-immunoprecipitations are indicated above. 1% of AIRE transfected whole-cell extract was loaded to control the expression of AIRE. Western blot was carried out with anti-AIRE (A) or anti-DNA-PKc (B) antibodies, positions of AIRE, cross-reacting IgG bands (A) and DNA-PK (B) are indicated left. Asterisk designates a nonspecific band occurring with anti-HA control antibody (B). (C) DNA-PK can interact with AIRE independently of DNA and RNA. For co-immunoprecipitations, either AIRE or control transfected HEK293 cells were used. Ethidium bromide (EtBr), micrococcal nuclease (MNase) and RnaseA/T mix treatment was performed where specified. Co-immunoprecipitations were carried out with anti-DNA-PK and anti-myc antibodies as indicated. Western blot was performed with anti-AIRE antibody, positions of AIRE and cross-reacting IgG bands are indicated left.

To further confirm the pull-down results and to demonstrate that the full-length AIRE protein is able to interact with DNA-PK, we carried out co-immunoprecipitation experiments. So far, no AIRE endogenous expression on protein level has been detected in immortalized cell-lines. As subcellular localization of transfected AIRE in immortalized cell lines is similar to the AIRE localization in thymus *in vivo*, as well as AIRE is able to activate transcription in those cells, we transfected HEK293 cells with the plasmid expressing full-length AIRE or empty vector as a negative control. The whole-cell extracts were prepared from transfected cells and used in co-immunoprecipitations with anti-DNA-PKcs, anti-AIRE or various control antibodies. The precipitates were separated on SDS-PAGE and immunoblotted with anti-AIRE antibody. As seen in Fig. 2A, anti-DNA-PKcs and anti-AIRE antibodies (as a positive control) were able to precipitate AIRE protein whereas control antibodies against GST, GAPDH and HuR (a nuclear mRNA-binding protein) could not. Similarly, AIRE and DNA-PKcs interaction was detectable when the immunoprecipitation with anti-AIRE antibody followed by the Western with anti-DNA-PKcs antibody was carried out (Fig. 2B).

Since the whole-cell extracts used in immunoprecipitations contained remarkable amounts of chromatin and RNA (data not shown), we next wanted to test whether the interaction between AIRE and DNA-PK is DNA and RNA dependent or independent. Previously, a DNA intercalator ethidium bromide (EtBr) has been shown to efficiently disrupt the binding of Ku proteins to Oct2 and DNA-PK [26,30], whereas the micrococcal nuclease has been widely used for the disruption of chromatin as well as for the digestion of contaminating DNA from cell lysates and immunoprecipitates [26,31]. Consequently, we transfected the HEK293 cells with the plasmid expressing full-length myc-tagged AIRE or empty vector as a negative control and performed co-immunoprecipitations with anti-DNA-PKcs antibodies in the presence of EtBr, RNaseA/T mix or treated the immunoprecipitates with the micrococcal nuclease (Fig. 2C, top panel). As a positive control, to show that the same level of the AIRE protein was present in each lysate, the immunoprecipitations were also performed with the anti-myc antibodies (Fig. 2C, bottom panel). Fig. 2C top panel reveals that DNA-PKcs still co-immunoprecipitates AIRE from the cell extracts treated with EtBr or RNaseA, which indicates that AIRE does not require DNA or RNA to interact with DNA-PKcs. However, when we treated the co-immunoprecipitates with micrococcal nuclease, we observed a weaker interaction between AIRE and DNA-PKcs (Fig. 2C, top panel), which suggests that in the cell lysates containing DNA and chromatin, a fraction of AIRE binds DNA-PK through the DNA and/or chromatin. In conclusion, the co-immunoprecipitation results together confirm that the AIRE protein interacts with DNA-PK.

### 3.3 DNA-PK phosphorylates the AIRE protein

**Fig. 3:**
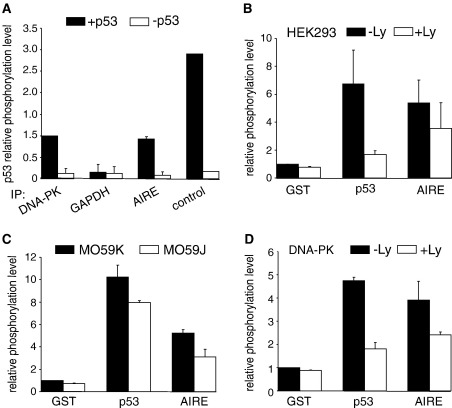
Phosphorylation of the AIRE protein *in vitro*. (A) AIRE co-immunoprecipitates kinase activity. GAPDH, DNA-PKcs and AIRE co-immunoprecipitates and nuclear extract (control) were used to carry out kinase reactions on p53 peptide or no substrate (−p53) as indicated. Relative phosphorylation level represents the relative amount of ^32^P that were incorporated into p53 peptide compared to phosphorylation level of p53 peptide achieved by DNA-PK immunoprecipitate (=1). The values (±standard errors) are means of two independent experiments, except for control where only single experiment was carried out. (B, C, D) Phosphorylation of GST-AIRE (AIRE). Kinase reactions were carried out using nuclear extracts prepared from HEK293 (B) or DNA-PKcs positive MO59K and negative MO59J cell-lines (C), or by purified DNA-PK. GST-p53 (p53) was used as a positive control, DNA-PK inhibitor LY294002 (Ly) was included if indicated (B, D). Phosphorylation levels are presented in relative values compared to phosphorylation level of GST (=1), the values (±standard errors) are means of at least two independent experiments.

DNA-PK is a nuclear serine/threonine protein kinase that has been reported to phosphorylate many nuclear targets. To study whether the AIRE protein is one of the targets of DNA-PK, we used several different assays which enabled quantitation of DNA-PK kinase activity. We first studied whether the AIRE protein co-immunoprecipitates DNA-PK kinase activity. Co-immunoprecipitations from nuclear extracts prepared from HEK293 cells transfected with myc-tagged AIRE were carried out with antibodies recognizing DNA-PKcs, myc tag and GAPDH (as a negative control). The co-immunoprecipitates were further incubated with biotinylated p53 derived peptide as a DNA-PK specific substrate [32] in the presence of DNA-PKcs activating dsDNA and ^32^P-γATP. As a positive control, HEK293 nuclear extract was used instead of the co-immunoprecipitates. Fig. 3A shows that a similar amount of ^32^P was incorporated into the p53 peptide when AIRE and DNA-PKcs co-immunoprecipitates were applied, whereas no activity was detectable with GAPDH co-immunoprecipitate. The relatively high phosphorylation efficiency achieved with AIRE co-immunoprecipitate was most likely due to the reason that the co-immunoprecipitation with anti-myc antibody is more efficient than that of with DNA-PK antibody. Since the p53 peptide used in the assay is a DNA-PK specific substrate [32], these data show that the AIRE/DNA-PK complex has kinase activity and suggest that AIRE, too, might be phosphorylated by DNA-PK.

To further investigate whether AIRE can be phosphorylated by DNA-PK, we carried out three different *in vitro* kinase assays where recombinant GST-AIRE, GST-p53 and GST alone were used as the substrates. First, we performed the experiments using the nuclear extract from HEK293 cells as a source of kinases in the absence or presence of a specific inhibitor for PI3 family kinases, LY294002 (Fig. 3B). Second, we did the kinase assay using the nuclear extracts either from DNA-PKcs positive (MO59K) or DNA-PKcs negative (MO59J) cell lines (Fig. 3C). Third, we carried out kinase reactions using a purified HeLa DNA-PK complex, again, in the absence or presence of LY294002 (Fig. 3D). As a result, we observed that the phosphorylation level of the AIRE protein reached up to 50–80% of that of the p53 protein in all three experimental approaches, whereas only background phosphorylation of GST alone was detected (Fig. 3B–D). In concordance, the kinase activity was decreased, although moderately, when LY294002 inhibitor was added (Fig. 3B) or when the nuclear extract from DNA-PKcs negative MO59J cells was used (Fig. 3C), indicating that besides DNA-PKcs other kinases can phosphorylate the AIRE protein.

**Fig. 4:**
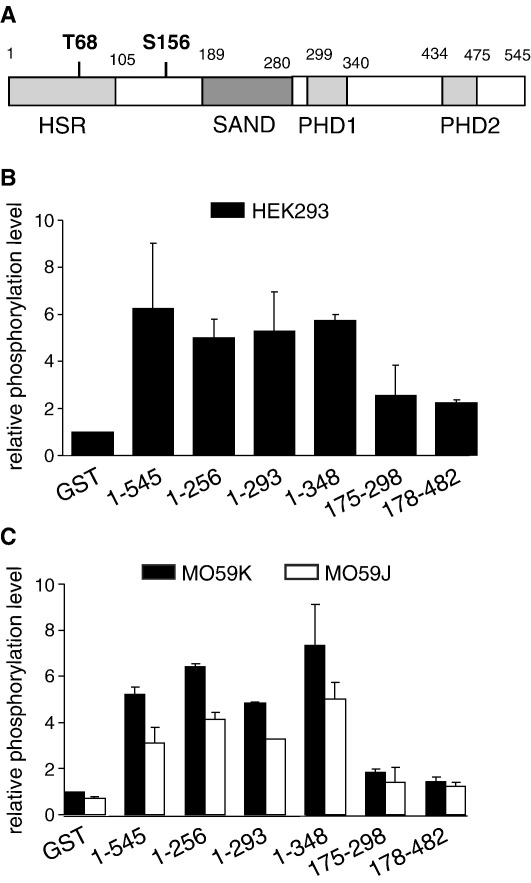
The N-terminal part of AIRE is a preferential target of phosphorylation. (A) Schematic representation of the AIRE protein domains. HSR (*h*omogenously *s*taining *r*egion), SAND (*S*p100, *A*IRE-1, *N*ucP41/75 and *D*EAF-1 potential DNA binding domain), PHD (*p*lant *h*omeo*d*omain zinc finger). (B, C) Phosphorylation of full-length (1–545) AIRE and the deletion mutants, the expressed amino acids are indicated. The kinase reactions were carried out using nuclear extracts prepared from HEK293 (B) or DNA-PKcs positive MO59K and negative MO59J cell lines (C). Phosphorylation levels are presented in relative values compared to phosphorylation level of GST (=1), the values (±standard errors) are means of at least two independent experiments.

In order to identify which region of the AIRE protein is phosphorylated, we expressed different AIRE domains as GST fusion proteins and tested their phosphorylation using the nuclear extracts as a source of kinases. The domain structure of the AIRE protein is given in Fig. 4A. Fig. 4B shows that the HEK293 nuclear extract phosphorylated more efficiently the GST-fusions of N-terminal AIRE containing the first 256 (1–256), 293 (1–293) or 348 (1–348) amino acids (Fig. 4B) while about 2 times less phosphorylation was seen with the GST-fusions of AIRE C-terminal fragments containing SAND (175–298) or SAND-PHD1-PHD2 (178–482) regions. A similar pattern of AIRE phosphorylation was observed when the phosphorylation assay was performed with DNA-PKcs competent MO59K or DNA-PKcs deficient MO59J cell extracts (Fig. 4C). Thus, although in pull-down experiments, the first PHD of AIRE alone was able to interact with DNA-PK, the main DNA-PK target seems to be the N-terminal part of AIRE indicating that AIRE and DNA-PK make multiple contacts. Taken together, these results demonstrate that DNA-PK can phosphorylate AIRE *in vitro* and that the N-terminal region of AIRE is the predominant target of DNA-PK.

### 3.4 T68 and S156 are the phosphorylation sites on the AIRE protein

**Fig. 5:**
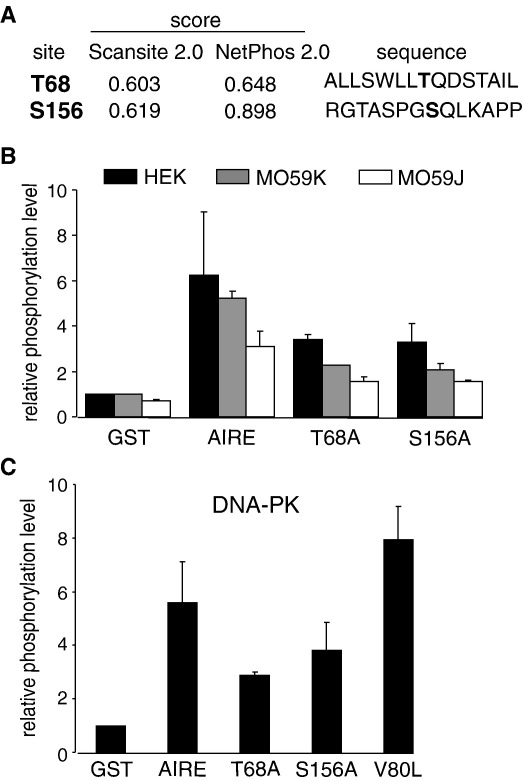
Amino acids Thr68 and Ser156 of AIRE are phosphorylated by DNA-PK. (A) Prediction of AIRE phosphorylation sites with Scansite 2.0 and NetPhos 2.0 programs. Score estimates importance of the prediction (from 0 to 1) whereas higher score indicates the higher confidence of the prediction. Sequence shows the context of the acceptor residue in bold ± 6–7 residues. (B, C) Influence of T68A and S156A mutations to AIRE phosphorylation efficiency. The kinase reactions were performed either by nuclear extracts prepared from HEK293, DNA-PKcs positive MO59K or negative MO59J cell-lines (B), or by purified DNA-PK (C). Phosphorylation levels are presented in relative values compared to phosphorylation level of GST (=1). The values (±standard errors) are means of at least two independent experiments (B, C). The GST-AIRE protein containing patient mutation V80L was used as a control (C).

The N-terminal region of AIRE contains the HSR domain that mediates di- or tetramerization of the protein [4,5]. Interestingly, previous *in vitro* experiments have indicated that AIRE dimerization may occur as a result of phosphorylation [4]. We next aimed to find the DNA-PK specific phosphorylation sites on AIRE. Using the Scansite 2.0 and NetPhos 2.0 programs, we found two putative DNA-PK phosphorylation sites: T68 within and S156 close to the HSR domain of the AIRE protein (Figs. 4A and 5A). To verify the prediction, we changed either Thr68 or Ser156 to alanines, both in wild type GST-AIRE and truncated GST-AIRE (1–256) constructs, and tested the influence of these mutations on the phosphorylation efficiency. Fig. 5B demonstrates that compared to GST-AIRE, the phosphorylation levels of the mutant proteins were decreased about 30–50% when the cell extracts from either HEK293 or MO59K cells were used as source of cellular kinases. Finally, we performed the phosphorylation assays using purified DNA-PK complex (Fig. 5C). In agreement with the previous experiments, a reduction of about 30% in the phosphorylation levels of the T68A and S156A mutants was detected, whereas the APECED patient mutation V80L did not negatively affect the AIRE phosphorylation level. Thus, we conclude that amino acids Thr68 and Ser156 of the AIRE protein are the phosphorylation sites of DNA-PK.

### 3.5 DNA-PK is expressed in thymic medullary epithelial cells (mTEC)

**Fig. 6:**
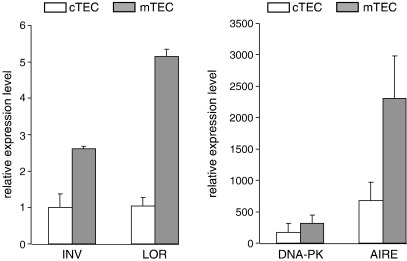
DNA-PK is expressed in mTEC cells. Thymuses were enzyme digested and FACS-sorted according to the expression of EpCAM and analyzed for the mRNA expression level of involucrin (INV), loricrin (LOR), DNA-PKcs and AIRE, using the quantitative RT-PCR. The relative expression levels are compared to involucrin level in cTEC cell population (=1), the values (±standard errors) are mean of two independent quantitative RT-PCR reactions both performed as triplicate measurements.

DNA-PK is a ubiquitously expressed protein which levels are enhanced by induction of DNA double strand brakes [17]. To study whether the DNA-PK and AIRE interaction might occur in vivo, we studied whether DNA-PK is also present in thymic medullary epithelial cells (mTEC): the only subset of cells where the AIRE protein expression is detected thus far [1–3]. Thus, we purified the thymic mTEC based on the cell surface marker EpCAM [29] and analyzed the expression of *Aire*, *DNA-PK* and two AIRE target genes; *involucrin* and *loricrin*. The *involucrin* and *loricrin* genes are highly expressed during the terminal differentiation of epidermal keratinocytes [33] and are downregulated in mTEC of Aire knock-out mouse [34]. Fig. 6, right panel demonstrates that DNA-PKcs mRNA is highly expressed in thymic epithelial cells, including the AIRE-enriched mTEC fraction. As characteristic to Aire-regulated genes in the thymus, the expression levels of *involucrin* and *loricrin* were relatively low, remarkable lower than those of *DNA-PKcs* and *Aire* in mTEC subpopulation (Fig. 6, left panel).

### 3.6 T68A and S156A mutations downregulate the transactivation activity of AIRE

**Fig. 7:**
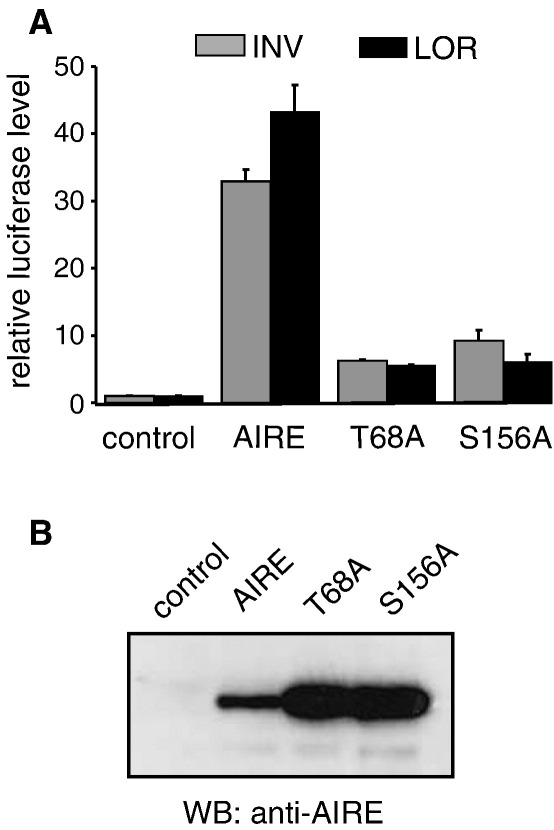
T68A and S156A mutations influence AIRE transactivation activity. (A) The HEK293 cells were transfected with pSI (control), pSI-AIRE (AIRE), pSI-AIRE-T68A (T68A), pSI-AIRE-S156A (S156A) and either INV-pBL (INV) or LOR-pBL (LOR) reporter promoters. Activations are presented in relative values compared to luciferase activity of the control (=1). The values are means (±standard errors) of two independent transfections. (B) The HEK293 cells were transfected with pSI (control), pSI-AIRE (AIRE), pSI-AIRE-T68A (T68A), pSI-AIRE-S156A (S156A) and analyzed by Western blotting with anti-AIRE antibody.

Previously, it has been demonstrated that several missense mutations in the HSR domain affect the dimerization, cellular localization and the transactivation ability of AIRE [13–15,35]. Thus, we next tested whether the mutation of AIRE phosphorylation sites influences the AIRE transactivation activity. We cloned AIRE with T68A and S156A mutations into mammalian expression vector and performed luciferase assays using the lysates from HEK293 cells transfected with wild type AIRE or the mutant constructs. As AIRE target genes, human *loricrin* and *involucrin* gene promoters fused to the luciferase gene were used. Fig. 7A demonstrates that when the HEK293 cells were transfected with AIRE T68A and AIRE S156A mutation constructs, significantly lower transactivation activity compared to the wild type AIRE was seen. Western blot of the transfected lysates with anti-AIRE antibody shows that both mutants are expressed even in higher level than the wild type AIRE construct (Fig. 7B). In conclusion, the luciferase assays suggest that the phosphorylation status of AIRE at T68 and S156 is important for the transactivation activity.

## 4 Discussion

In this study, we demonstrate that AIRE interacts with and is phosphorylated by the DNA-PK complex. The best DNA-PK substrates are the DNA associated proteins and the most effective phosphorylation occurs when DNA-PK is colocalized with the same DNA molecule as its target proteins [36]. Binding to the Ku proteins improves the affinity of DNA-PKcs for DNA ends about 100-fold [37]. This is in keeping with the finding showing that AIRE is able to bind DNA via its SAND domain [4]. In addition to the double strand brakes, the DNA-PK and/or the Ku proteins are known to bind telomeres and sequence-specific promoter elements [17,24], single stranded DNA [38], nucleosomes [39], RNA [18] and base unpairing regions (BURs) which typically are found in nuclear matrix attachment regions (MARs) [40]. Ku70 and Ku80 also colocalize to RNA polymerase II elongation sites [41,42]. Interestingly, AIRE has been reported to associate with nuclear matrix [43] and has been proposed to regulate gene clusters [44]. In addition, the AIRE protein contains PHD domains, which recently have been shown to recognize specifically modified histones within the chromatin [10,11]. Thus, AIRE appears to be a chromatin and DNA-binding protein. In this study, we observed that although AIRE is able to bind DNA-PK without DNA, most likely the DNA or chromatin is contributing to the AIRE and DNA-PK interaction (Fig. 2C). This is in concordance with DNA-PK ability to phosphorylate its substrates more efficiently at the presence of DNA [17]. At least two possibilities can be considered how DNA-PK, AIRE and DNA or chromatin may interact. First, DNA or chromatin, or even certain DNA sequence elements or specifically modified chromatin, may increase the affinity of AIRE for DNA-PK. Second, posttranslationally modified AIRE subpopulations may bind DNA-PK in different conditions. As we cannot exclude that this type of experiments can be influenced by the overexpression of AIRE, further studies are needed to find out the role of chromatin in AIRE and DNA-PK interaction *in vivo,* especially in the thymic environment.

Phosphorylation of AIRE by cAMP dependent protein kinases A (PKA) and C (PKC) has been reported earlier [4], though, the phosphorylated domain of AIRE remained unknown. We here demonstrate that *in vitro* the AIRE protein is mainly phosphorylated at the N-terminal region and define two residues, Thr68 and Ser156, as DNA-PK specific target sites. Thr68 resides within and Ser156 is located not far from the HSR domain. Both these phosphorylation sites are located outside the PHD1 finger, the domain of AIRE that was initially shown to pull-down DNA-PK, indicating that multiple different contacts can occur between AIRE and the DNA-PK complex. However, as previously reported [4], our *in vitro* phosphorylation studies also indicate that other kinases may phosphorylate the AIRE protein, and that phosphorylation sites other than Thr68 and Ser156 should exist within AIRE.

The HSR domain of AIRE has been shown to be responsible for the dimerization and tetramerization of the protein [4,5] and several, but not all, APECED mutations within the HSR decrease AIRE transactivation activity and influence AIRE cellular localization [15,16]. In addition, previous *in vitro* experiments have indicated that AIRE dimerization may occur as a result of phosphorylation [4]. As shown in this study, conversion of Thr68 and Ser156 amino acids to alanines suppresses AIRE transactivation activity. However, we did not observe any influence of these mutations to AIRE dimerization and cellular localization (data not shown). Thus, since the mutations that do not impact AIRE dimerization and the dot formation ability can downregulate AIRE transactivation activity, the N-terminal part of AIRE appears to be involved in transcriptional regulation not only via its dimerization activity. It is possible that status of AIRE phosphorylation at N-terminus influences AIRE interactions with other protein partners or with DNA.

In response to ionizing radiation, DNA-PK can have indirect effects on transcriptional regulation. It has been shown that phosphorylation of interferon regulated factor 3 (IRF-3) and pancreatic duodenal homeobox-1 (PDX-1) protein by DNA-PK in response to ionizing radiation directs these proteins to degradation by the proteasome and therefore downregulates IRF-3 and PDX-1 target genes [45,46]. Alternatively, phosphorylation of transcription factor Oct-1 by DNA-PK in response to ionizing radiation leads to stabilization but disrupts transactivation activity of Oct-1. As a result, the expression of Oct-1 target genes U6 and Histone H2B is downregulated [47]. We also studied whether UV irradiation changes AIRE cellular localization and expression level in various cell cultures but we could not detect remarkable differences (data not shown). Thus, it seems that DNA-PK phosphorylates AIRE independently of its function in DNA repair.

Based on research on AIRE deficient mice, it is evident that AIRE upregulates the expression level of many genes in thymus [48,49]. Studies in tissue culture cells confirm that AIRE indeed activates several promoters [5,13,15,16]. Thus, although *in vivo* the AIRE protein expression is only detected in thymic medullary epithelial cells [1–3], the transfected AIRE protein has similar transactivating properties, and nuclear localization pattern as AIRE in the thymus [14,16]. Despite these studies, the mechanisms of how AIRE regulates its target genes are still unclear. Posttranscriptional modifications, like phosphorylation, acetylation and ubiquitination, are widely used to regulate activity of transcription factors and regulators. In this study, we first describe the phosphorylation sites of AIRE and propose that its phosphorylation status is important for its transactivation activity. However, several questions remain, including what are the signals that trigger AIRE phosphorylation and how AIRE phosphorylation is connected with its function in the thymus. The differentiation of AIRE positive thymic epithelial cells was recently shown to be dependent on RANK-mediated signaling [50], which is known to activate several protein kinases [51]. As we showed in this study, DNA-PK is expressed in medullary epithelial cells (Fig. 6). Whether the thymic epithelial differentiation process correlates with the activation of DNA-PK and other possible AIRE-specific kinases needs further studies.

## Figures and Tables

**Table 1:** Mass spectrometry analysis of GST-PHD1 binding proteins

SDS-PAGE sample (kDa)	MALDI/TOF peptide mass map analysis			MALDI-TOF/TOF fragment analysis	
	Protein identity	Theoretical mass (kDa)	Sequence coverage% (number of peptides)		

Exp1	> 250	DNA-PKcs	473.8	11.9 (43)	ATQQQHDFTLTQTADGR
	80	Ku80	83.1	5.6 (5)	IKLFPLIEAK KKDQVTAQEIFQDNHEDGPTAK
	70	Ku70	70.1	22.3 (14)	n.d.
Exp2	> 250	DNA-PKcs	473.8	15.7 (57)	n.d.
	80	Ku80	83.1	24.1 (17)	n.d.
	70	Ku70	70.1	16.4 (9)	n.d.

Two different experiments (exp1 and exp2) were carried out, n.d. means not determined.

## References

[1] Finnish-German Consortium (1997). An autoimmune disease, APECED, caused by mutations in a novel gene featuring two PHD-type zinc-finger domains. Nat. Genet..

[2] Nagamine K., Peterson P., Scott H.S., Kudoh J., Minoshima S., Heino M., Krohn K.J., Lalioti M.D., Mullis P.E., Antonarakis S.E., Kawasaki K., Asakawa S., Ito F., Shimizu N. (1997). Positional cloning of the APECED gene. Nat. Genet..

[3] Heino M., Peterson P., Kudoh J., Nagamine K., Lagerstedt A., Ovod V., Ranki A., Rantala I., Nieminen M., Tuukkanen J., Scott H.S., Antonarakis S.E., Shimizu N., Krohn K. (1999). Autoimmune regulator is expressed in the cells regulating immune tolerance in thymus medulla. Biochem. Biophys. Res. Commun..

[4] Kumar P.G., Laloraya M., Wang C.Y., Ruan Q.G., Davoodi-Semiromi A., Kao K.J., She J.X. (2001). The autoimmune regulator (AIRE) is a DNA-binding protein. J. Biol. Chem..

[5] Pitkanen J., Doucas V., Sternsdorf T., Nakajima T., Aratani S., Jensen K., Will H., Vahamurto P., Ollila J., Vihinen M., Scott H.S., Antonarakis S.E., Kudoh J., Shimizu N., Krohn K., Peterson P. (2000). The autoimmune regulator protein has transcriptional transactivating properties and interacts with the common coactivator CREB-binding protein. J. Biol. Chem..

[6] Bottomley M.J., Collard M.W., Huggenvik J.I., Liu Z., Gibson T.J., Sattler M. (2001). The SAND domain structure defines a novel DNA-binding fold in transcriptional regulation. Nat. Struct. Biol..

[7] Bienz M. (2006). The PHD finger, a nuclear protein-interaction domain. Trends Biochem. Sci..

[8] Gozani O., Karuman P., Jones D.R., Ivanov D., Cha J., Lugovskoy A.A., Baird C.L., Zhu H., Field S.J., Lessnick S.L., Villasenor J., Mehrotra B., Chen J., Rao V.R., Brugge J.S., Ferguson C.G., Payrastre B., Myszka D.G., Cantley L.C., Wagner G., Divecha N., Prestwich G.D., Yuan J. (2003). The PHD finger of the chromatin-associated protein ING2 functions as a nuclear phosphoinositide receptor. Cell.

[9] Schultz D.C., Friedman J.R., Rauscher F.J. (2001). Targeting histone deacetylase complexes via KRAB-zinc finger proteins: the PHD and bromodomains of KAP-1 form a cooperative unit that recruits a novel isoform of the Mi-2alpha subunit of NuRD. Genes Dev..

[10] Shi X., Hong T., Walter K.L., Ewalt M., Michishita E., Hung T., Carney D., Pena P., Lan F., Kaadige M.R., Lacoste N., Cayrou C., Davrazou F., Saha A., Cairns B.R., Ayer D.E., Kutateladze T.G., Shi Y., Cote J., Chua K.F., Gozani O. (2006). ING2 PHD domain links histone H3 lysine 4 methylation to active gene repression. Nature.

[11] Wysocka J., Swigut T., Xiao H., Milne T.A., Kwon S.Y., Landry J., Kauer M., Tackett A.J., Chait B.T., Badenhorst P., Wu C., Allis C.D. (2006). A PHD finger of NURF couples histone H3 lysine 4 trimethylation with chromatin remodelling. Nature.

[12] Bottomley M.J., Stier G., Pennacchini D., Legube G., Simon B., Akhtar A., Sattler M., Musco G. (2005). NMR structure of the first PHD finger of autoimmune regulator protein (AIRE1). Insights into autoimmune polyendocrinopathy-candidiasis-ectodermal dystrophy (APECED) disease. J. Biol. Chem..

[13] Björses P., Halonen M., Palvimo J.J., Kolmer M., Aaltonen J., Ellonen P., Perheentupa J., Ulmanen I., Peltonen L. (2000). Mutations in the AIRE gene: effects on subcellular location and transactivation function of the autoimmune polyendocrinopathy-candidiasis-ectodermal dystrophy protein. Am. J. Hum. Genet..

[14] Pitkanen J., Vahamurto P., Krohn K., Peterson P. (2001). Subcellular localization of the autoimmune regulator protein. Characterization of nuclear targeting and transcriptional activation domain. J. Biol. Chem..

[15] Halonen M., Kangas H., Ruppell T., Ilmarinen T., Ollila J., Kolmer M., Vihinen M., Palvimo J., Saarela J., Ulmanen I., Eskelin P. (2004). APECED-causing mutations in AIRE reveal the functional domains of the protein. Human Mutat..

[16] Pitkanen J., Rebane A., Rowell J., Murumagi A., Strobel P., Moll K., Saare M., Heikkila J., Doucas V., Marx A., Peterson P. (2005). Cooperative activation of transcription by autoimmune regulator AIRE and CBP. Biochem. Biophys. Res. Commun..

[17] Smith G.C., Jackson S.P. (1999). The DNA-dependent protein kinase. Genes Dev..

[18] Zhang S., Schlott B., Gorlach M., Grosse F. (2004). DNA-dependent protein kinase (DNA-PK) phosphorylates nuclear DNA helicase II/RNA helicase A and hnRNP proteins in an RNA-dependent manner. Nucleic Acids Res..

[19] Koike M. (2002). Dimerization, translocation and localization of Ku70 and Ku80 proteins. J. Radiat. Res..

[20] Giffin W., Kwast-Welfeld J., Rodda D.J., Prefontaine G.G., Traykova-Andonova M., Zhang Y., Weigel N.L., Lefebvre Y.A., Hache R.J. (1997). Sequence-specific DNA binding and transcription factor phosphorylation by Ku Autoantigen/DNA-dependent protein kinase. Phosphorylation of Ser-527 of the rat glucocorticoid receptor. J. Biol. Chem..

[21] Sartorius C.A., Takimoto G.S., Richer J.K., Tung L., Horwitz K.B. (2000). Association of the Ku autoantigen/DNA-dependent protein kinase holoenzyme and poly(ADP-ribose) polymerase with the DNA binding domain of progesterone receptors. J. Mol. Endocrinol..

[22] Mayeur G.L., Kung W.J., Martinez A., Izumiya C., Chen D.J., Kung H.J. (2005). Ku is a novel transcriptional recycling coactivator of the androgen receptor in prostate cancer cells. J. Biol. Chem..

[23] Giffin W., Gong W., Schild-Poulter C., Hache R.J. (1999). Ku antigen-DNA conformation determines the activation of DNA-dependent protein kinase and DNA sequence-directed repression of mouse mammary tumor virus transcription. Mol. Cell. Biol..

[24] Xu P., LaVallee P.A., Lin J.J., Hoidal J.R. (2004). Characterization of proteins binding to E-box/Ku86 sites and function of Ku86 in transcriptional regulation of the human xanthine oxidoreductase gene. J. Biol. Chem..

[25] Lee K.A.W., Zerivitz K., Akusjärvi G., celis J.E. (1994). Cell Biology: A Laboratory Handbook, Academic Press.

[26] Lai J.S., Herr W. (1992). Ethidium bromide provides a simple tool for identifying genuine DNA-independent protein associations. Proc. Natl. Acad. Sci. U. S. A..

[27] Obenauer J.C., Cantley L.C., Yaffe M.B. (2003). Scansite 2.0: Proteome-wide prediction of cell signaling interactions using short sequence motifs. Nucleic Acids Res..

[28] Blom N., Gammeltoft S., Brunak S. (1999). Sequence and structure-based prediction of eukaryotic protein phosphorylation sites. J. Mol. Biol..

[29] Kont V., Laan M., Kisand K., Merits A., Scott H.S., Peterson P. (2008). Modulation of Aire regulates the expression of tissue-restricted antigens. Mol. Immunol..

[30] Hsu H.L., Yannone S.M., Chen D.J. (2002). Defining interactions between DNA-PK and ligase IV/XRCC4. DNA Repair (Amst).

[31] Nguyen T.N., Goodrich J.A. (2006). Protein–protein interaction assays: eliminating false positive interactions. Nat. Methods.

[32] Lees-Miller S.P., Sakaguchi K., Ullrich S.J., Appella E., Anderson C.W. (1992). Human DNA-activated protein kinase phosphorylates serines 15 and 37 in the amino-terminal transactivation domain of human p53. Mol. Cell. Biol..

[33] Ishida-Yamamoto A., Kartasova T., Matsuo S., Kuroki T., Iizuka H. (1997). Involucrin and SPRR are synthesized sequentially in differentiating cultured epidermal cells. J. Invest. Dermatol..

[34] Derbinski J., Gabler J., Brors B., Tierling S., Jonnakuty S., Hergenhahn M., Peltonen L., Walter J., Kyewski B. (2005). Promiscuous gene expression in thymic epithelial cells is regulated at multiple levels. J. Exp. Med..

[35] Ramsey C., Bukrinsky A., Peltonen L. (2002). Systematic mutagenesis of the functional domains of AIRE reveals their role in intracellular targeting. Hum. Mol. Genet..

[36] Gottlieb T.M., Jackson S.P. (1993). The DNA-dependent protein kinase: requirement for DNA ends and association with Ku antigen. Cell.

[37] Hammarsten O., Chu G. (1998). DNA-dependent protein kinase: DNA binding and activation in the absence of Ku. Proc. Natl. Acad. Sci. U. S. A..

[38] Torrance H., Giffin W., Rodda D.J., Pope L., Hache R.J. (1998). Sequence-specific binding of Ku autoantigen to single-stranded DNA. J. Biol. Chem..

[39] Park E.J., Chan D.W., Park J.H., Oettinger M.A., Kwon J. (2003). DNA-PK is activated by nucleosomes and phosphorylates H2AX within the nucleosomes in an acetylation-dependent manner. Nucleic Acids Res..

[40] Galande S., Kohwi-Shigematsu T. (1999). Poly(ADP-ribose) polymerase and Ku autoantigen form a complex and synergistically bind to matrix attachment sequences. J. Biol. Chem..

[41] Lomberk G., Bensi D., Fernandez-Zapico M.E., Urrutia R. (2006). Evidence for the existence of an HP1-mediated subcode within the histone code. Nat. Cell Biol..

[42] Mo X., Dynan W.S. (2002). Subnuclear localization of Ku protein: functional association with RNA polymerase II elongation sites. Mol. Cell. Biol..

[43] Akiyoshi H., Hatakeyama S., Pitkanen J., Mouri Y., Doucas V., Kudoh J., Tsurugaya K., Uchida D., Matsushima A., Oshikawa K., Nakayama K.I., Shimizu N., Peterson P., Matsumoto M. (2004). Subcellular expression of autoimmune regulator is organized in a spatiotemporal manner. J. Biol. Chem..

[44] Johnnidis J.B., Venanzi E.S., Taxman D.J., Ting J.P., Benoist C.O., Mathis D.J. (2005). Chromosomal clustering of genes controlled by the aire transcription factor. Proc. Natl. Acad. Sci. U. S. A..

[45] Karpova A.Y., Trost M., Murray J.M., Cantley L.C., Howley P.M. (2002). Interferon regulatory factor-3 is an in vivo target of DNA-PK. Proc. Natl. Acad. Sci. U. S. A..

[46] Lebrun P., Montminy M.R., Van Obberghen E. (2005). Regulation of the pancreatic duodenal homeobox-1 protein by DNA-dependent protein kinase. J. Biol. Chem..

[47] Schild-Poulter C., Shih A., Yarymowich N.C., Hache R.J. (2003). Down-regulation of histone H2B by DNA-dependent protein kinase in response to DNA damage through modulation of octamer transcription factor 1. Cancer Res..

[48] Anderson M.S., Venanzi E.S., Klein L., Chen Z., Berzins S.P., Turley S.J., von Boehmer H., Bronson R., Dierich A., Benoist C., Mathis D. (2002). Projection of an immunological self shadow within the thymus by the Aire protein. Science.

[49] Liston A., Lesage S., Wilson J., Peltonen L., Goodnow C.C. (2003). Aire regulates negative selection of organ-specific T cells. Nat. Immunol..

[50] Rossi S.W., Kim M.Y., Leibbrandt A., Parnell S.M., Jenkinson W.E., Glanville S.H., McConnell F.M., Scott H.S., Penninger J.M., Jenkinson E.J., Lane P.J., Anderson G. (2007). RANK signals from CD4(+)3(−) inducer cells regulate development of Aire-expressing epithelial cells in the thymic medulla. J. Exp. Med..

[51] Wada T., Nakashima T., Hiroshi N., Penninger J.M. (2006). RANKL-RANK signaling in osteoclastogenesis and bone disease. Trends Mol. Med..

